# Driving Pressure During General Anesthesia for Open Abdominal Surgery (DESIGNATION): study protocol of a randomized clinical trial

**DOI:** 10.1186/s13063-020-4075-z

**Published:** 2020-02-18

**Authors:** The DESIGNATION–investigators, Liselotte Hol, Sunny G. L. H. Nijbroek, Ary Serpa Neto, Marcelo Gama de Abreu, Paolo Pelosi, Sabrine N. T. Hemmes, Leon P. H. J. Aarts, Ronald D. L. Akkerman, Juliette J. E. Albersen, Caterina Aurilio, Denise Battaglini, Hans D. de Boer, Annemieke Boom, Christa Boer, Tammo Brouwer, Wolfgang F. F. A. Buhre, Carolina S. E. Bulte, Gitara M. Edward-Rutten, Marc B. Godfried, Hendrik J. F. Helmerhorst, Jan Hofland, Hester Hoogenboom, W. Ten Hoope, Bernard M. Houweling, Ragnar Huhn, Wanda Konijn, Ankie W. M. M. Koopman–van Gemert, Dianne J. de Korte-de Boer, Minke C. Kortekaas, Felix van Lier, Benedikt Preckel, Mandana Rad, Pasquale Sansone, André Stamkot, Robert Jan Stolker, Bram Thiel, Johannes F. H. Ubben, Michel M. R. F. Struys, Bastiaan A. in ‘t Veld, Hermann Wrigge, Miriam Zeillemaker-Hoekstra, Tim van der Zwan, Johannes H. M. J. Zwijsen, Markus W. Hollmann, Marcus J. Schultz

**Affiliations:** Department of Anesthesiology, Amsterdam University Medical Centers, location Academic Medical Center, Meibergdreef 9, 1105 AZ Amsterdam, The Netherlands

**Keywords:** Mechanical ventilation, Intraoperative ventilation, ΔP, Compliance, Positive end-expiratory pressure, Recruitment maneuver, Pulmonary complications, Postoperative complications, Postoperative pulmonary complications

## Abstract

**Background:**

Intraoperative driving pressure (ΔP) is associated with development of postoperative pulmonary complications (PPC). When tidal volume (V_T_) is kept constant, ΔP may change according to positive end-expiratory pressure (PEEP)-induced changes in lung aeration. ΔP may decrease if PEEP leads to a recruitment of collapsed lung tissue but will increase if PEEP mainly causes pulmonary overdistension. This study tests the hypothesis that individualized high PEEP, when compared to fixed low PEEP, protects against PPC in patients undergoing open abdominal surgery.

**Methods:**

The “Driving prESsure durIng GeNeral AnesThesIa for Open abdomiNal surgery trial” (DESIGNATION) is an international, multicenter, two-group, double-blind randomized clinical superiority trial. A total of 1468 patients will be randomly assigned to one of the two intraoperative ventilation strategies. Investigators screen patients aged ≥ 18 years and with a body mass index ≤ 40 kg/m^2^, scheduled for open abdominal surgery and at risk for PPC. Patients either receive an intraoperative ventilation strategy with individualized high PEEP with recruitment maneuvers (RM) (“individualized high PEEP”) or one in which PEEP of 5 cm H_2_O without RM is used (“low PEEP”). In the “individualized high PEEP” group, PEEP is set at the level at which ΔP is lowest. In both groups of the trial, V_T_ is kept at 8 mL/kg predicted body weight. The primary endpoint is the occurrence of PPC, recorded as a collapsed composite of adverse pulmonary events.

**Discussion:**

DESIGNATION will be the first randomized clinical trial that is adequately powered to compare the effects of individualized high PEEP with RM versus fixed low PEEP without RM on the occurrence of PPC after open abdominal surgery. The results of DESIGNATION will support anesthesiologists in their decisions regarding PEEP settings during open abdominal surgery.

**Trial registration:**

Clinicaltrials.gov, NCT03884543. Registered on 21 March 2019.

## Background

Postoperative pulmonary complications (PPC) occur frequently in patients undergoing major surgery [1] and their development is associated with perioperative mortality and morbidity [2]. Patients undergoing open abdominal surgery are particularly at risk of developing PPC, according to a high “Assess Respiratory Risk in Surgical Patients in Catalonia for PPC” (ARISCAT) score [3, 4]. Given that an estimated 55 million abdominal surgical procedures are performed each year worldwide [1, 5], even a small reduction in the occurrence of PPC would have a large impact on healthcare and costs.

Mechanical ventilation is an essential intervention during general anesthesia for surgery, but carries a risk of injuring lung tissue, leading to PPC. Intraoperative ventilation is one of the modifiable risk factors associated with the development of PPC [6, 7]. Cyclic lung recruitment and overdistension are two main mechanisms responsible for the injurious effects of ventilation [8]. Intraoperative lung-protective ventilation with a low tidal volume (V_T_) combined with high positive end-expiratory pressure (PEEP) with recruitment maneuvers (RM) prevents against PPC [2], but the precise role of high PEEP and RM herein is highly uncertain. Three randomized clinical trials (RCTs) showed intraoperative ventilation with low V_T_ plus high PEEP and RM to prevent against PPC when compared with ventilation with high V_T_ plus low PEEP without RM [9–11]. Two other RCTs, however, showed no benefit of high PEEP with RM compared to low PEEP without RM, when V_T_ was kept low [12, 13].

It has been suggested that high PEEP should be titrated so that it reduces cyclic recruitment, while at the same time preventing overdistension [14]. An individualized high PEEP strategy, avoiding an increase in driving pressure (ΔP), or a decrease in respiratory system compliance (C_RS_), may better protect against PPC than one that uses fixed high PEEP. Until now, only two RCTs have tested this hypothesis. One RCT in patients undergoing intraoperative ventilation for major abdominal surgery did not show a benefit of individualized high PEEP and RM for the primary endpoint of development of postoperative complications. However, the individualized PEEP strategy did reduce PPC, one of the secondary endpoints of that study [15]. Another RCT, in patients undergoing intraoperative one-lung ventilation for thoracic surgery, also showed an individualized high PEEP strategy to protect against PPC [16], albeit at a low fragility index [17]. Thus, the results of these two RCTs should be interpreted with caution.

The aim of the “Driving prESsure durIng GeNeral AnesThesIa for Open abdomiNal surgery trial” (DESIGNATION) is to compare intraoperative ventilation with individualized high PEEP and RM versus ventilation with a fixed low PEEP without RM in patients planned for open abdominal surgery with respect to the development of PPC. We hypothesize that the individualized high PEEP strategy protects against PPC in this surgical population.

## Methods

### Objectives and design

**Fig. 1:**
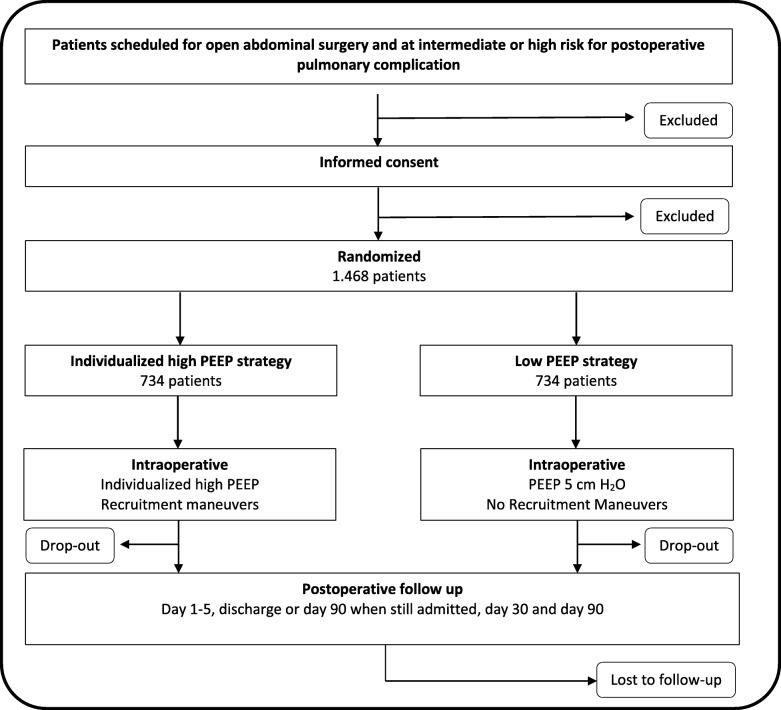
Consolidated Standards of Reporting Trials (CONSORT) diagram for the “Driving prESsure durIng GeNeral AnesThesIa for Open abdomiNal surgery” (DESIGNATION) trial

DESIGNATION is an international, multicenter, prospective, two-group, double-blind RCT in patients planned for open abdominal surgery and at risk of developing PPC. A total of 1468 patients will be recruited in 22 hospitals (Appendix 1) and randomly assigned to one of the two to be tested intraoperative ventilation strategies (see Consolidated Standards of Reporting Trials diagram in Fig. 1).

DESIGNATION tests the premise that, in patients planned for open abdominal surgery and at risk for PPC, an individualized high PEEP strategy that aims at reducing atelectasis but preventing overdistension better protects against the development of PPC than a strategy that uses a fixed high PEEP.

The trial has been approved by the Institutional Review Board (IRB) of the Amsterdam University Medical Centers, location “Academic Medical Center,” Amsterdam, the Netherlands. DESIGNATION was registered at clinicaltrials.gov on 21 March 2019 (study identifier NCT03884543).

### Study population

Local investigators screen patients aged ≥ 18 years with a maximum body mass index (BMI) of 40 kg/m^2^ planned for open abdominal surgery. The number of patients meeting these enrollment criteria is recorded by means of a screening log file. Patients are eligible if they are at intermediate to high risk for PPC according to the ARISCAT score (Table 1) [3].

**Table 1:** Assess Respiratory Risk in Surgical Patients in Catalonia scores

Risk for PPC of variables selected for the logistic regression model			
	Multivariate analysis	Β coefficients	Risk score
	OR (95% CI) *n* = 1625		
Age (years)			
≤ 50	1		
51–80	1.4 (0.6–3.3)	0.331	3
> 80	5.1 (1.9–13.3)	1.619	16
Preoperative SpO_2_ (%)			
≥ 96	1		
91–95	2.2 (1.2–4.2)	0.802	8
≤ 90	10.7 (4.1–28.1)	2.375	24
Respiratory infection in last month	5.5 (2.6–11.5)	1.698	17
Preoperative anemia (≤ 10 g/dL)	3.0 (1.4–6.5)	1.105	11
Surgical incision			
Peripheral	1		
Upper abdominal	4.4 (2.3–8.5)	1.480	15
Intrathoracic	11.4 (4.9–26.0)	2.431	24
Duration of surgery (h)			
≤ 2	1		
> 2–3	4.9 (2.4–10.1)	1.593	16
> 3	9.7 (4.7–19.9)	2.268	23
Emergency procedure	2.2 (1.04–4.5)	0.768	8

SpO_2_ oxyhemoglobin saturation by pulse oximetry breathing air in supine position

High or intermediate risk for postoperative pulmonary complications following abdominal surgery: ARISCAT risk score ≥ 26

Patients planned for laparoscopic surgery, for open abdominal surgery combined with intra-thoracic surgery, or for surgery in prone or lateral positions are excluded from participation. Patients with a reported pregnancy, patients who consented for another interventional study, patients who did not have sufficient reflection time before giving informed consent (i.e. < 12 h), and patients declining to participate in DESIGNATION are not eligible. Patients who have received mechanical ventilation for > 30 min within the last 30 days before the current surgery, patients who are expected to require postoperative ventilation in the intensive care unit (ICU) or post-anesthesia care unit, patients with persistent hemodynamic instability or intractable shock, and patients with severe cardiac disease (New York Heart Association class [NYHA] III or IV, or acute coronary syndrome [ACS], or persistent ventricular tachyarrhythmias) are excluded. Other exclusion criteria are: any major previous lung surgery (e.g. lung resection); (previous) acute respiratory distress syndrome (ARDS); history of previous severe chronic obstructive pulmonary disease (COPD) GOLD III or IV or with (non-invasive) ventilation and/or oxygen therapy at home; and no written informed consent.

### Standard ventilation management

Patients in both groups are ventilated in volume-controlled mode, at the lowest possible inspired oxygen fraction (FiO_2_), but at least 0.4, to maintain oxygen saturation > 90%. Inspiratory to expiratory ratio (I:E) is set at 1:2 and the respiratory rate is adjusted to allow for normocapnia (end-tidal carbon dioxide partial pressure in the range of 35–45 mmHg [4.6–5.9 kPa]). Tidal volume is set at 8 mL/kg predicted body weight (PBW), where PBW is calculated according to a predefined formula: 50 + 0.91 × (centimeters of height – 152.4) for men and 45.5 + 0.91 × (centimeters of height – 152.4) for women [18].

### Interventions

Patients assigned to the “individualized high PEEP” group undergo a RM before and after the “decremental PEEP trial,” after any disconnection from the ventilator and within 1 h before extubation. RM are only performed after assuring a stable hemodynamic situation, as judged by the attending anesthesiologist. During each RM the ventilator remains in a volume-controlled ventilation mode, with the respiratory rate set at 15 breaths/min. In steps of 15 s, PEEP is increased from 5 cm H_2_O in steps of 5 cm H_2_O up to 20 cm H_2_O.

**Fig. 2:**
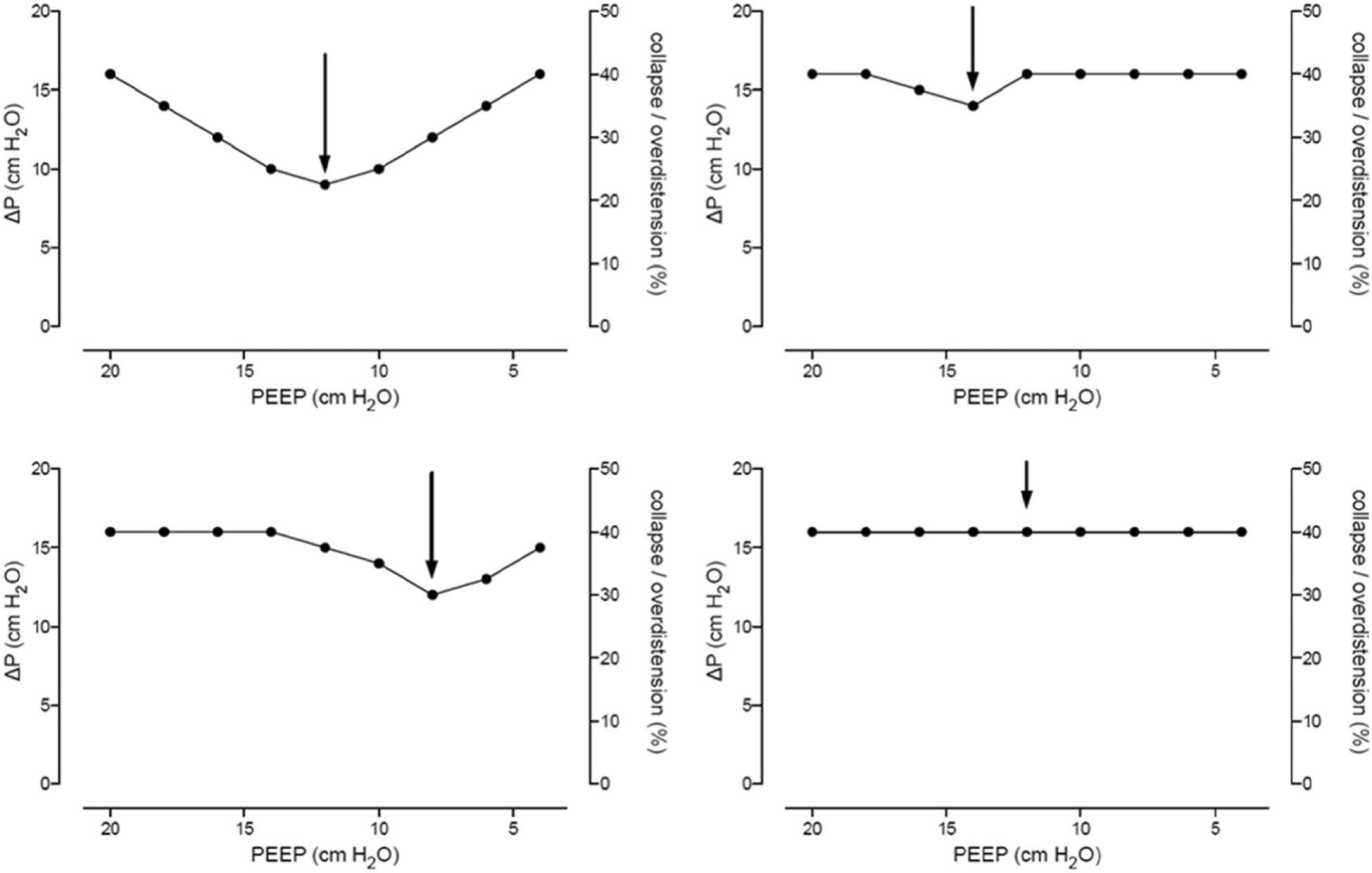
Examples of the “ΔP–PEEP” graph. The arrow represents the optimal PEEP

The decremental PEEP trial starts at the end of the first RM at a PEEP of 20 cm H_2_O with the respiratory rate set at 15 breaths/min. During the decremental PEEP trial, the ventilator remains in volume-controlled ventilation mode. Every 20 s, PEEP is decreased in steps of 2 cm H_2_O till a minimum level of 6 cm H_2_O. With every step, the resulting ΔP is calculated by subtracting PEEP from the plateau pressure (P_plat_) at the end of each step. ΔP is plotted against PEEP to construct a bedside “ΔP–PEEP” graph, as shown in Fig. 2. From the ΔP–PEEP graph, the highest level of PEEP with the lowest ΔP is selected. If no nadir in ΔP is observed in the ΔP–PEEP graph, 12 cm H_2_O PEEP will be used.

**Fig. 3:**
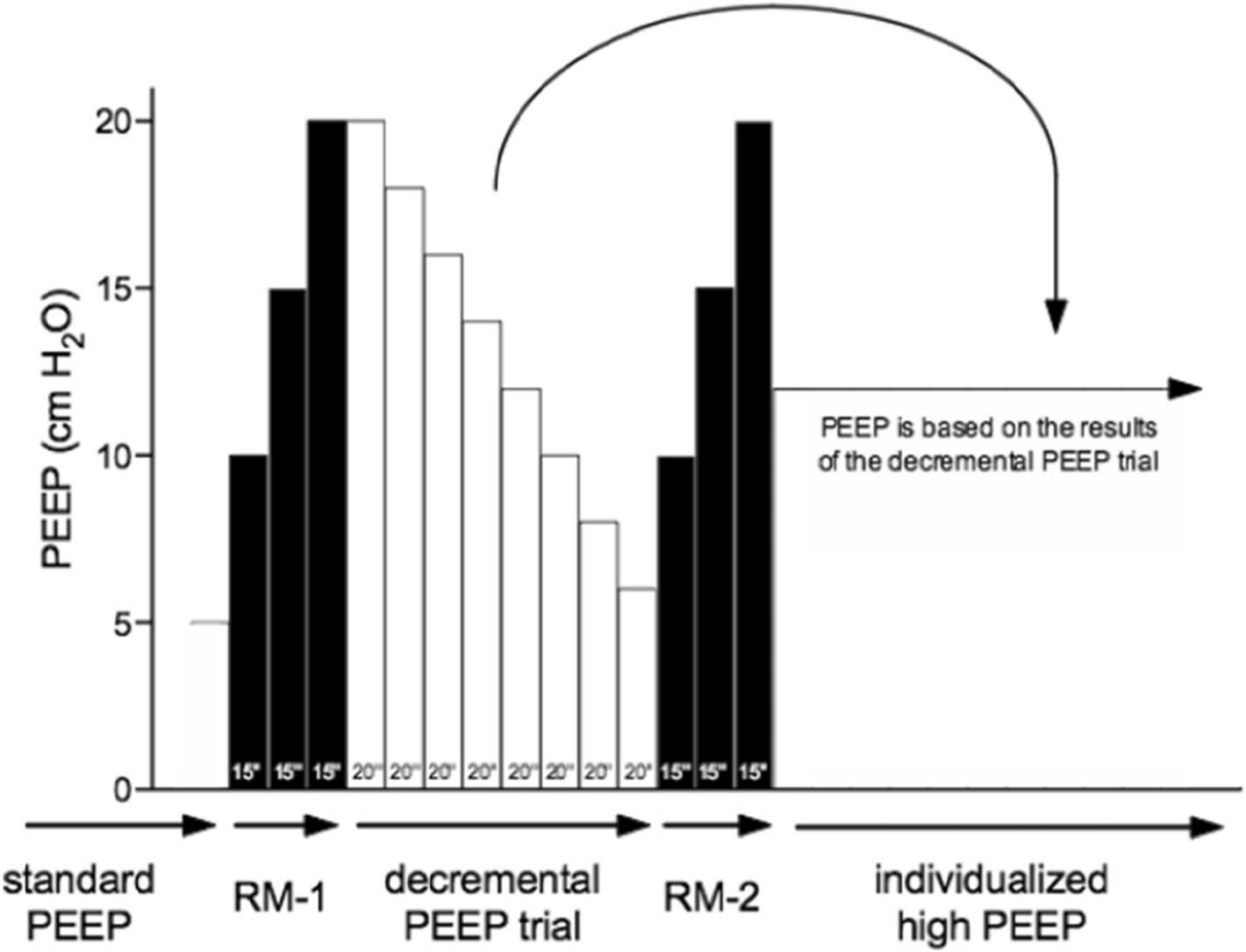
Overview of the intervention: the recruitment maneuvers and decremental PEEP trial. See text for a detailed description of the recruitment maneuvers and the decremental PEEP trial. The numbers projected in each bar represent the duration of each step in seconds

The decremental PEEP trial is followed by a second RM, after which PEEP is set and kept at the level at which ΔP is lowest, as indicated by the decremental PEEP trial (Fig. 3).

Patients assigned to the “low PEEP” group are ventilated with a fixed 5 cm H_2_O PEEP for the complete duration of intraoperative ventilation. They will neither receive RM nor a decremental PEEP trial.

### Rescue therapies

In the “individualized high PEEP” group, desaturation (defined as SpO_2_ ≤ 90% or if preoperative SpO_2_ < 90% an absolute decrease in SpO_2_ > 5%) may still suggest overdistension, despite a low ΔP. If desaturations in the absence of airway problems, severe hemodynamic impairment, or ventilator malfunction in this group occurs, a rescue strategy is allowed in which PEEP is stepwise decreased and eventually FiO_2_ is increased (Table 2).

**Table 2:** Rescue for desaturation in the “individualized high PEEP” group

Rescue for desaturation		
Step	PEEP	FiO_2_
1	20	0.4
2	18	0.4
3	16	0.4
4	14	0.4
5	12	0.4
6	12	0.5
7	12	0.6
8	10	0.6
9	8	0.6
10	6	0.6
11	6	0.7
12	6	0.8

Down-titration of PEEP as rescue of desaturation. Starts at the level of PEEP set after the decremental PEEP trial

In the “low PEEP” group, atelectasis may cause desaturations. If desaturations in the absence of airway problems, severe hemodynamic impairment, or ventilator malfunction in this group occurs, a rescue strategy is allowed where FiO_2_ is increased first, eventually followed by RM and PEEP increases (Table 3).

**Table 3:** Rescue for desaturation in the “low PEEP” group

Rescue for desaturation		
Step	PEEP	FiO_2_
1	5	0.4
2	5	0.5
3	5	0.6
4	5	0.7
5	5	0.8
6	6	0.8
7	RM	

Up-titration of PEEP and recruitment maneuvers (RM) as rescue of desaturation

If there is any concern about the patient’s safety or in the unlikely event that PEEP interferes with the surgical procedure, the ventilation settings can be changed at any time. PEEP can be changed according to the decision by the anesthesiologist in charge if any of the following pre-approved reasons for protocol deviations occur: hypotension, defined as a decrease in mean arterial pressure (MAP) > 20% compared to preoperative MAP and lasting > 3 min, not responding to intravenous fluids and/or vasoactive drugs; new arrhythmias not responding to the treatment suggested by the Advanced Cardiac Life Support Guidelines [19]; need for vasoactive drugs at dosages higher than acceptable according to the discretion of the anesthesiologist in charge; need for massive transfusion, with > 5 units of blood to maintain hematocrit > 21% (or hemoglobin > 7 mg/dL); and surgical complications determining life-threatening situations. Any deviation from the protocol, other than those mentioned above, are considered protocol violations. Protocol violations are to be reported and will be discussed with the Data Safety Monitoring Board (DSMB).

### Standard procedures beyond ventilator management

To avoid interference with the trial intervention, routine general anesthesia, postoperative pain management, physiotherapeutic procedures, and fluid management will be used in the intraoperative and/or postoperative period according to each center’s specific expertise and routine clinical practice. However, adherence to the enhanced recovery after surgery (ERAS) guidelines is strongly recommended. These guidelines advise to perform postoperative pain management in order to achieve a visual analog scale (VAS) pain score < 4 and to use regional analgesia whenever indicated. Furthermore, the guidelines suggest using physiotherapy by early mobilization, deep breathing exercise with and without incentive spirometry, and stimulation of cough in the postoperative period. The guidelines recommend avoiding fluid overload (according to the discretion of the attending anesthesiologist) and to use appropriate prophylactic antibiotics when indicated. Quantitative neuromuscular monitoring (e.g. train-of-four [TOF]) is required. Residual curarization should be excluded before extubation (e.g. TOF > 0.9).

### Minimization of bias

Randomization is performed by the local investigators using a dedicated and password protected randomization module in Research Electronic Data Capture (REDCap). Included patients will be randomly allocated in a 1:1 ratio to the “individualized high PEEP” group or the “low PEEP” group. The allocation sequence is computer-generated and stratified per center and by BMI (≤ 30 vs > 30 kg/m^2^). Permuted blocks of different block sizes are used, with a maximum block size of eight.

At each site, at least two investigators are involved with the study. One researcher will perform the randomization directly before surgery and is responsible for intraoperative ventilation and data collection. A second investigator will remain blinded for the randomization arm and is responsible for the postoperative data collection.

The surgeon and patient are kept blind to the allocated study intervention. Since PEEP can be adjusted at any time point upon the surgeon’s request or because of concerns about patient’s safety, and since patients do not profit from knowing to which group they are allocated to, unblinding is not applicable.

### Study endpoints

The primary endpoint of DESIGNATION is a composite of PPC occurring in the first five postoperative days. This endpoint follows the European Perioperative Clinical Outcome (EPCO) definition and has been used before in several RCTs [12, 15, 20].

The composite consists of the following PPC:

(i) severe respiratory failure, defined as need for non-invasive or invasive mechanical ventilation, or a PaO_2_ < 60 mmHg (or < 7.9 kPa) or SpO_2_ < 90% despite supplemental oxygen in spontaneously breathing patients;
(ii) bronchospasm, defined as newly detected expiratory wheezing treated with bronchodilators;
(iii) suspected pulmonary infection defined as receiving antibiotics and meeting at least one of the following criteria: new or changed sputum, new or changed lung opacities on chest radiograph when clinically indicated, tympanic temperature > 38.3 °C, or white blood cell count > 12,000/μL;
(iv) pulmonary infiltrate, defined as any unilateral or bilateral infiltrates on chest radiography;
(v) aspiration pneumonitis, defined as respiratory failure after the inhalation of regurgitated gastric contents;
(vi) atelectasis, defined as lung opacification with shift of the mediastinum, hilum, or hemidiaphragm towards the affected area, and compensatory overinflation in the adjacent non-atelectasis lung on chest radiography;
(vii) ARDS, according to the Berlin definition for ARDS [21];
(viii) pleural effusion, defined as blunting of the costophrenic angle, loss of the sharp silhouette of the ipsilateral hemidiaphragm in upright position, evidence of displacement of adjacent anatomical structures, or (in supine position) a hazy opacity in one hemithorax with preserved vascular shadows on chest radiography;
(ix) cardiopulmonary edema, defined as clinical signs of congestion, including dyspnea, edema, rales and jugular venous distention, with the chest radiograph demonstrating increase in vascular markings and diffuse alveolar interstitial infiltrates; and
(x) pneumothorax, defined as air in the pleural space with no vascular bed surrounding the visceral pleura on chest radiography.

Secondary endpoints include mild respiratory failure, defined as a PaO_2_ < 60 mmHg (or < 7.9 kPa) or SpO_2_ < 90% in room air, but responding to supplemental oxygen (excluding hypoventilation); intraoperative complications that are not related to induction or change of depth of anesthesia and are scored during steady state, including: desaturation (SpO_2_ ≤ 90% or if preoperative SpO_2_ < 90% an absolute decrease in SpO_2_ > 5% and lasting > 1 min), hypotension (decrease in MAP of > 20% and lasting for > 3 min), any need for vasoactive agents defined as more than needed to compensate for vasodilating effects of anesthesia, and any new arrhythmias needing intervention as suggested by the Advanced Cardiac Life Support Guidelines [19]); intraoperative fluid strategy; impaired wound healing; rate of all-cause mortality and in-hospital mortality; unplanned admission to an ICU; length of ICU (if applicable) and hospital stay; and postoperative extrapulmonary complications including sepsis (according to the SEPSIS-3 definition [22]), septic shock (defined as sepsis with persisting hypotension requiring vasopressors to maintain MAP ≥ 65 mmHg and having a serum lactate level of > 2 mmol/L despite adequate fluid resuscitation) [22], extrapulmonary infections (wound infection + any other infection), anastomotic leakage, and acute renal failure (as defined by the Acute Kidney Injury Network [AKIN] [23]).

### Study visits and data collection

Patients are seen before surgery and followed during surgery and daily during the first five postoperative days or until hospital discharge, whatever occurs first. If patients remain hospitalized after the first five postoperative days, an extra visit at hospital discharge will be performed. Day 90 is defined as the last day of follow-up; accordingly, patients still admitted to the hospital will be last contacted 90 days after surgery. Patients are contacted by telephone around day 30 and day 90 (Appendix 2).

Patients will be recruited at the anesthesiology or surgical outpatient clinic, or at the surgical ward. Individuals will be informed verbally by local researchers and by a patient information letter (Additional file 2). The patient will be given sufficient time to consider their decision and to discuss the decision with their relatives or the independent expert.

During the preoperative visit, after signing the informed consent form, baseline variables are collected, including age, gender, height, weight, American Society of Anesthesiologist (ASA) score, functional status (independent, partially dependent, or totally independent), cardiac status (hearth failure according to the NYHA, ACS, or persistent ventricular tachyarrhythmias), and smoking status. Pulmonary status, COPD, including inhalation therapy and/or systemic steroids, respiratory infection within the last month, and mechanical ventilation for > 30 min in the last 30 days will be collected as well. Other baseline variables are: history of active cancer; weight loss > 10% in the last six months; history of diabetes mellitus and the use of insulin or oral anti-diabetics; type of scheduled surgery (emergency, urgent, or elective surgery); surgical procedure; transfusion of blood products in the last 6 h; and vital parameters (tympanic temperature, respiratory rate, SpO_2_, blood pressure, VAS score for pain during breathing, and heart rate). Blood tests (HbA1C, glucose, urea, creatinine, hemoglobin, white blood cell count) and chest imaging (assessed on mono- and bilateral infiltrate, pleural effusion, atelectasis, pneumothorax, and cardiopulmonary edema) are collected, but only if deemed necessary for clinical care for the patient.

During the intraoperative period, variables are recorded after induction, after RM, and hourly during anesthesia. The intraoperative variables to be collected are: ventilator settings; vital parameters; fluid and transfusion requirements; type and dose of administered vasoactive drugs; need of rescue therapy for desaturation; intraoperative complications possibly related to PEEP titrations; protocol deviations and violations; duration of surgery; and duration and details of anesthesia (type of anesthesia, volatile versus total intravenous anesthesia [TIVA] versus combined, epidural analgesia, neuromuscular function monitoring). For the patients assigned to the “individualized high PEEP” group, the plateau pressure will be documented at the end of each step of the decremental PEEP trial. The bedside-constructed “ΔP–PEEP graph” will be saved in the electronic database.

Postoperatively, clinical data and the presence of pulmonary and extrapulmonary postoperative complications are collected. Blood test and chest imaging will only be taken if deemed necessary for clinical care of the patients. Life status (death or alive) will be scored at the first five postoperative days, on day 30 and day 90. Length of stay in hospital, unplanned admission to an ICU, and length of stay in the ICU are collected around day 90.

### Study dropouts and missing data

Participation in the DESIGNATION trial is voluntary. The number of drop-outs is expected to be very low. The patient can withdraw consent for collecting study data and for participation in the study at any time during the trial and without giving a reason for this. To minimize patient drop-out due to cancellation of surgery, randomization will be performed on the day of surgery just before induction of anesthesia. Drop-out patients will not be replaced. No or minimal losses to follow-up for the primary and secondary outcomes are anticipated. Lost to follow-up cases due to withdrawal of consent or for other reasons will be excluded form analysis. If > 1% of missing data were found for the primary outcome, a sensitivity analysis using multiple imputations and estimating-equation methods will be carried out.

### Handling of data

Patient identifying personal data will be replaced by an assigned code. Directly identifying data will only be used to contact the patients and are only available for the local investigators. Handling of personal data complies with the general data protection regulations and applicable national regulations. Data will be collected in an electronic case report form (CRF) stored in REDCap. REDcap is a password-protected, secure, web-based application, which includes validation checks, audit trails, and appropriate use access rights. After completing the data collection, full access to the database will be granted to selected investigators. Data are restricted, available after analyses and publication of the main paper. All data will be stored in a secure place for 15 years after study end.

The results of DESIGNATION will be published in scientific journals and used for national and international guideline. A summary of the results will be placed on clinicaltrials.gov to inform participants.

### Sample size calculation

The required sample size is calculated based on an estimated effect size derived from individual patient data form previous clinical trials [9–12, 24]. A total of 720 patients per group are required, assuming a two-sided significance level of 0.05 and a power of 80% to detect the expected difference in the primary outcome between a control group proportion of 34% and an experimental group proportion of 27.2% (relative risk reduction of 20%). To allow for a dropout rate of 2% (based on comparable RCTs of intraoperative ventilation performed by our group), 734 patients per group (a total of 1468) are needed.

### Statistical analysis

The detailed statistical analysis plan will be updated, finalized, and made available before the inclusion of the last patient.

All statistical analyses will be conducted according to the modified intention-to-treat principle considering all patients in the treatment groups to which they were randomly assigned, excluding cases lost to follow-up due to withdrawal of consent or cancellation of surgery. Hypothesis tests will be two-sided with a significance level of 5% for all outcomes. Patient and baseline characteristics will be compared and described by appropriate statistics.

The effects of the intervention on the primary outcome, occurrence of any PPC, will be reported as number and percentages and estimated with risk ratio and 95% confidence intervals (CI) calculated with Wald’s likelihood ratio approximation test and with χ^2^ tests for hypothesis testing. Kaplan–Meier curves will be used to report time to PPC, and hazard ratios with a 95% CI will be calculated with Cox proportional hazard models without adjustment for covariates. The Schoenfeld residuals against the transformed time will be used to test the proportional hazard assumptions and alternative parametric survival models will be used if the proportionality assumption is not sustained.

Treatment effects on incidence of PPC will be analyzed according to the following subgroups: (i) age < 65 years versus ≥ 65 years; (ii) BMI < 30 kg/m^2^ versus BMI ≥ 30 kg/m^2^; (iii) baseline SpO_2_ < 96% versus SpO_2_ ≥ 96%; (iv) moderate versus high risk for PPC; (v) duration of surgery < 3 h versus ≥ 3 h; and (6) planned destination to ICU or HDU versus ward. Analyses of heterogeneity of effects across subgroups will performed with the use of treatment-by-subgroup interaction term, added to a generalized linear model considering a binomial distribution, and will be presented in a forest plot.

The effect of the intervention on other binary outcomes will be assessed with risk ratio and 95% CIs calculated with Wald’s likelihood ratio approximation test and with χ^2^ tests for hypothesis testing. Time-to-event data will be assessed using Kaplan–Meier curves, and hazard ratios with a 95% CI will be calculated with Cox proportional hazard models without adjustment for covariates. The Schoenfeld residuals against the transformed time will be used to test the proportional hazard assumptions and alternative parametric survival models will be used if the proportionality assumption is not sustained.

The effects of the intervention on length of hospitalization and ICU stay will be estimated with generalized linear models considering distributions that will fit a possible heavy right-tailed distribution without zero (such as truncated Poisson, gamma distribution, or inverse Gaussian), choosing the best fit according to the model’s deviance.

In all analyses, statistical uncertainties will be quantified with two-sided 95% CIs. A two-sided *p* value < 0.05 will be considered statistically significant. Additional sensitivity analyses will be reported in the detailed statistical analysis plan. Data analysis will be performed blinded for the allocated study intervention.

### Trial organization

The steering committee consists of the two principal investigators, two trial coordinators, four international experts on intraoperative ventilation who contributed to the design of the study protocol, and the local investigators at participating study sites. The steering committee remains responsible for the interpretation of the data and drafts the final report that will be approved by all investigators.

An independent DSMB, consisting of four anesthesiologists with extensive clinical research experience (Daniel Sessler [chair], Jennifer Hunter, Jeanine Wiener–Kronisch, and Klaus Markstaller) will overview study conduct and possible side effects of the study treatment at 25%, 50%, and 75% of patient inclusions. A statistician will report to the DSMB. All study-related or possibly study-related (serious) adverse events (AEs) will be reported to the DSMB. AEs are defined as any undesirable experience occurring to a subject during the study, whether or not considered related to the experimental intervention. A serious AE (SAE) is any toward medical occurrence or effect that results in death, is life-threatening, requires hospitalization or prolongation of existing inpatients’ hospitalization, results in persistent or significant disability or incapacity, is a congenital anomaly or birth defect, or any other important medical event that did not result in any of the outcomes listed above due to medical or surgical intervention but could have been based upon appropriate judgement by the investigator. SAEs related to the intervention will be directly reported to the IRB. AEs related or possibly related to the intervention and SAEs not related to the intervention will be reported to the IRB in a line-listing.

This study is an investigator-initiated trial, funded by “The Netherlands Organization for health Research and Development” (ZonMw) and sponsored by the Amsterdam University Medical Centers, location AMC. The sponsor can suspend the study if there are sufficient grounds that continuation of the study will jeopardize the individuals’ health or safety.

An independent monitor will perform clinical trial monitoring according to the monitor plan. Remote monitoring will be performed to signal early aberrant patterns, issues with consistency, credibility, and other anomalies. On-site monitoring will comprise controlling presence and completeness of the research dossier and the informed consent forms, and source data check will be performed as described in the monitoring plan. Centralized initiation meetings will be organized before sites can start including patients.

A complete checklist of recommended items to address in a clinical trial protocol and related documents according to the “Standard Protocol Items: Recommendations for Interventional Trials (SPIRIT) 2013” is provided (see Additional file 1).

## Discussion

DESIGNATION tests the hypothesis that an “individualized high PEEP” strategy, titrated to the level at which ΔP is lowest, protects against development of PPC.

Mechanical ventilation is mandatory during general anesthesia but has the potential to induce lung injury promoting the development of PPC. Positive pressure ventilation can result in overdistension of the aerated parts of the heterogeneously aerated lung. Non- or poorly aerated parts of the lung are prone for cyclic collapse and recruitment. Both overdistension as well as cyclic recruitment contributes to inflammation and injury. Locally produced inflammatory mediators could even leak to the circulation, eventually causing extrapulmonary, or distal organ failure [7, 8].

In DESIGNATION, the primary endpoint is a collapsed composite of PPC [20]. This endpoint has been used before in several clinical trials of intraoperative ventilation [12, 13, 15]. PPC can sensibly be combined as they share common pathophysiological mechanism. Each PPC seems to increase the risk of mortality and length of hospital stay in surgical patients [4]. This makes the composite of PPC a clinically relevant outcome measure. Furthermore, using a composite outcome measure increases the power of the study. In DESIGNATION, both the composite endpoint as the incidence of each PPC will be reported separately.

DESIGNATION also allows for determining the impact of the intraoperative ventilation strategy on the occurrence of extrapulmonary complications and on the length of stay in hospital and ICU. These endpoints are not only seen as clinically relevant but are also needed to understand the associated healthcare costs.

One meta-analyses demonstrated a lung-protective effect of intraoperative ventilation with low tidal volumes [25]. Therefore, all participants in DESIGNATION will receive ventilation with a low tidal volume (i.e. 8 mL/kg predicted bodyweight). This is in line with current recommendations [25–27] and is used in previous clinical trials of intraoperative ventilation before [12, 13, 15], allowing for good comparison of the DESIGNATION results with those from preceding trials.

Although the beneficial effect of ventilation with a low tidal volume is well established, the exact role of PEEP remains controversial [9, 11–13, 28]. Two RCTs showed that intraoperative ventilation with high PEEP does not protect against the development of PPC when tidal volumes are kept low [12, 13]. It had been suggested that high PEEP should be titrated according to the lung’s compliance or driving pressure. In the DESIGNATION trial, the level of PEEP that results in the lowest ΔP will be identified by the decremental PEEP trial. While in one previous trial of individualized PEEP, an incremental PEEP trial was used to find the best level of PEEP [16], here a decremental PEEP trial is chosen to ascertain that the resulting PEEP sustains the beneficial effects of the RM before the PEEP trial. This cannot be guaranteed when using an incremental PEEP trial, because PEEP is most likely below the closing pressure at start of an incremental PEEP trial. This could theoretically result in re-collapsing of certain lung parts [29], reversing the effect of the preceding RM.

The results of one systematic review and meta-analysis suggest that a change in PEEP resulting in an increase of ΔP increases the risk for PPC [14]. Previous trials of individualized PEEP did not use the PEEP resulting in the lowest ΔP, or the highest compliance, but PEEP set several cm H_2_O above the level that resulted in the lowest ΔP [15, 30]. This approach, however, may very well result in additional overdistension, and is therefore not followed in DESIGNATION. Indeed, in DESIGNATION, PEEP is set to the exact level at which ΔP is the lowest. If no nadir in the ΔP curve is present, PEEP will be set at 12 cm H_2_O. This level is chosen because clinical studies have shown PEEP of 12 cm H_2_O to result in maximum lung opening throughout intraoperative ventilation, irrespective of the FiO_2_ [12, 31–35].

In DESIGNATION, throughout the whole period of intraoperative ventilation, PEEP in the “individualized high PEEP” group is set according to the results of the “ΔP–PEEP graph” constructed soon after induction of anesthesia. Although it is possible that lung mechanics change during the surgical procedure, e.g. by manipulations of abdominal organs by the surgeon, or a change in position of the patient from a Trendelenburg position to an anti-Trendelenburg position and vice versa, it is decided not to repeat the decremental PEEP trial during surgery. Since the surgical intervention might interference with the result of the PEEP titration, a reliable decremental PEEP trial can only be performed if the surgeon temporarily stops the surgical intervention. This would consume too much time and is considered not feasible for this trial and clinical practice itself. DESIGNATION is a pragmatic study, and since ΔP is not expected to change comprehensively intraoperatively, it is decided not to repeat the PEEP titrations during surgery for the purpose of this study. Per design of the decremental PEEP trial, the individualized high PEEP will only result in PEEP in the range of 6–20 cm H_2_O. Nevertheless, 6 cm H_2_O PEEP can still result in overdistension. Based on the results of previous investigations, the optimal PEEP level is unlikely to be < 6 cm H_2_O [15, 30]. Therefore, the expected amount of overdistension in DESIGNATION will be negligible.

Patients in the “low PEEP” group receive ventilation with PEEP 5 cm H_2_O. The decision to use a PEEP of 5 cm H_2_O derives from previous trials of intraoperative ventilation [15, 16]. PEEP 5 cm H_2_O is the most frequently chosen level in daily practice [1, 36]; although there is no absolute consensus on the best level of PEEP, the overall recommendation is to ventilate patients with healthy lungs with this PEEP level.

Patients in the “individualized high PEEP” group will undergo at least three RMs during intraoperative ventilation. RMs are performed by increases in PEEP during a fixed time interval. This RM can be performed with all types of anesthesia ventilators, ensuring standardization of RM across different participating centers [15]. The stepwise approach has shown to be effective in increasing lung compliance and oxygenation without interruption of mechanical ventilation [11]. Furthermore, it compares favorably to other types of RM, such as “bag squeezing,” with respect to hemodynamic side effects [28]. In contrast to patients in the “individualized high PEEP” group, patients in the “low PEEP” group, will not receive a RM. RM are added to the “individualized high PEEP” group because applying PEEP without a RM has proven to be ineffective in reducing atelectasis [37]. Of note, in current daily practice, RM are not performed so often [1]. To have a group of patients that received ventilation representative for what is done in daily practice, it was decided not to perform RM in the control group. To avoid de-recruitment of the lungs, the RM will be repeated in the “individualized high PEEP” group after disconnection of the ventilator and within the last hour of anesthesia.

To protect patients but minimize the interference with the allocated PEEP level, rescue therapies are allowed. To the best interest of patients, pre-approved protocol deviations are permitted. Since these rescue therapies and deviations influence the clinical applicability of the “individualized high PEEP” strategy, both the use of protocol deviations as rescue strategies will be collected. Perioperative care will be performed to each center specific expertise and routine clinical use. However, to minimize inference of clinical care on the study intervention, it is suggested adhering to the ERAS guidelines. In DESIGNATION, patients in the “individualized high PEEP” group will receive a higher intraoperative PEEP compared to the patients in the “low PEEP” group. Previous research showed higher PEEP levels to result in more hypotension and higher need for vasoactive drugs [12, 13]. To monitor the impact of mechanical ventilation strategy on arterial blood pressure, intraoperative oxygenation, and respiratory system mechanics, variables regarding respiratory function and hemodynamics will be collected hourly during anesthesia. The DSMB, consisting of four members with expertise in clinical research, is instituted to overview the study conduct and possible side effects.

DESIGNATION aims at minimizing bias by using concealed allocation and blinding of the outcome assessor. REDcap, a secure web-based electronic password protected system, is used as database and randomization tool. REDcap randomization allocation sequence is computer-generated using a permuted block of different sizes. To minimize bias, randomization is stratified per center. Since obesity is an important factor influencing patient’s respiratory function during general anesthesia [38–40], randomization is also stratified by BMI (≤ 30 vs > 30 kg/m^2^).

DESIGNATION will be performed in both community and teaching hospitals in different countries of Europe, making the results generalizable. Intra-abdominal hypertension during a laparoscopic surgical procedure will have a considerable impact on lung mechanics [41–43]. For this reason, patients planned to undergo a laparoscopic surgical procedure are excluded from participation in DESIGNATION, even if the procedure is only laparoscopic assisted. There is a clear trend from open to laparoscopic surgery, and probably the number open abdominal procedures will decrease significantly. If DESIGNATION shows an individualized high PEEP strategy to be beneficial, additional trials testing the effects of personalized PEEP on occurrence of PPC will be needed.

In summary, DESIGNATION will be the first sufficiently powered, multicenter RCT to test whether a ventilation strategy of individualized high PEEP with RM is superior to one that uses fixed low PEEP of 5 cm H_2_O without RM with respect to development of PPC in patients planned for open abdominal surgery. The results of DESIGNATION will support anesthesiologists in their decision to set intraoperative PEEP during protective ventilation for general anesthesia during open abdominal surgery.

## Trial status

The current approved version of the protocol is version 4.2, issue data: 11 December 2019. DESIGNATION trial is currently recruiting patients. Recruitment started in April 2019 and will be completed in approximately April 2022.

### Medical terminology (in order of appearance)

Driving pressure: the pressure that results from moving air into the lungs; driving pressure is calculated by distracting the PEEP from plateau airway pressure *or* by dividing tidal volume by respiratory system compliance.

Postoperative pulmonary complications: spectrum of complications affecting the respiratory system and developing after ventilation for general anesthesia during surgery.

Tidal volume: the volume of air moved into and out of the lungs during each ventilation cycle.

Positive end-expiratory pressure: positive pressure during expiration, meant to keep maintain the airway pressure above the atmospheric level.

Open abdominal surgery: surgical procedure performed in the abdominal region requiring a large incision; laparoscopic devices are not used.

Cyclic lung recruitment: repetitive collapse and re-expansion of atelectatic lung tissue during each breath.

Overdistension: excessive stretching of (parts of) the lungs.

Recruitment maneuver: procedure is used for reinflation of collapsed lung units.

Respiratory system compliance: change in volume of the lungs for each unit increase in airway pressure; in other words; the ease with which the lungs can be inflated.

“High PEEP”: an applied PEEP level of > 5 cm H_2_O.

Volume-controlled (ventilation) mode: the artificial breath is delivered at a constant and predetermined inspiratory flow-time profile, with a fixed tidal volume.

Inspired oxygen fraction: the volumetric fraction of oxygen in the inhaled gas.

End-tidal carbon dioxide partial pressure: concentration of carbon dioxide at the end of exhalation.

Predicted bodyweight: the ideal body weight, calculated based on patients’ gender and height; used to normalize tidal volume to lung size.

Desaturation: situation in which the percentage of hemoglobin bindings sites in the bloodstream occupied by oxygen gets < 90%.

Train-of-four: a peripheral nerve simulation test, used to assess neuromuscular transmission when neuromuscular blocking agents are given to block musculoskeletal activity.

Volatile anesthesia: technique of general anesthesia that uses inhalation anesthetics.

Total intravenous anesthesia: technique of general anesthesia that uses a combination of drugs given exclusively by the intravenous route, i.e. without use of volatile anesthesia.

Collapsed composite endpoint: endpoint that is a combination of multiple clinical endpoints; endpoints are combined because they are considered to be of similar importance, occur with about the same frequency, have similar relative risk reductions, and have similar biologic mechanisms.

Incremental PEEP trial: progressively increasing PEEP with a constant tidal volume.

Decremental PEEP trial: starting at a high PEEP and progressively reducing it with a constant tidal volume.

Trendelenburg position: variant of the supine position in which the table is tilted with the head down and feet up.

Anti-Trendelenburg: variant of the supine position in which the table is tilted with the head up and feet down.

Bag squeezing: a type of recruitment maneuver performed manually by using an anesthesia bag.

## Supplementary information

**Additional file 1.** The SPIRIT checklist.

**Additional file 2.** Model consent form, version 4.0 (11–10-19).

## Figures and Tables

**Fig. 4:**
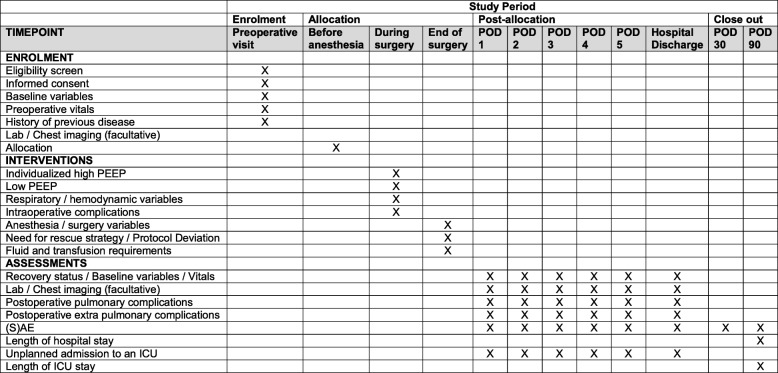
Schedule for enrolment and assessments (SPIRIT 2013 figure). *ICU* intensive care unit, *PEEP* positive end-expiratory pressure, *POD* postoperative day

## Data Availability

The datasets analyzed during the present study are available from the corresponding author on a reasonable request*.*

## Appendix 1

**Table 4:** DESIGNATION investigators

Site ID	Site name	Collaborator(s)
104	Department of Anesthesiology, Albert Schweitzer Hospital, Dordrecht, the Netherlands	Stamkot, André
		Kortekaas, Minke C.
		Koopman – van Gemert, Ankie W.M.M.
114	Department of Anesthesiology, Amsterdam University Medical Center location AMC, Amsterdam, the Netherlands	Hollmann, Markus W.
		Schultz, Marcus J.
		Preckel, Benedikt
		Hemmes, Sabrine N.T.
		Serpa Neto, Ary
		Nijbroek, Sunny G.L.H.
		Hol, Liselotte
97	Department of Anesthesiology, Amsterdam University Medical Center location VUmc, Amsterdam, the Netherlands	Bulte, Carolina S.E.
		Albersen, Juliette J.E
		Boer, Christa
118	Department of Anesthesiology, Bergmannstrost Klinikum, Halle, Germany	Wrigge, Hermann
101	Department of Anesthesiology, Bernhoven Hospital, Uden, the Netherlands	Hoogenboom, Hester
107	Department of Anesthesiology, Erasmus Medical Center, Rotterdam, the Netherlands	Van Lier, Felix
		Stolker, Robert Jan
103	Department of Anesthesiology, Haaglanden Medical Center in The Hague, the Netherlands	In ‘t Veld, Bastiaan A.
		Houweling, Bernard M.
102	Department of Anesthesiology, HAGA Hospital, The Hague, the Netherlands	Van der Zwan, Tim
		Rad, Mandana
117	Department of Anesthesiology, IRCCS Ospedale Policlinic San Martino, Genoa, Italy	Pelosi, Paolo
		Battaglini, Denise
105	Department of Anesthesiology, Leiden University Medical Center, Leiden, the Netherlands	Aarts, Leon P.H.J.
		Helmerhorst, Hendrik J.F.
		Akkerman, Ronald D.L.
113	Department of Anesthesiology, Maastricht University Medical Center, Maastricht, the Netherlands	Ubben, Johannes F.H.
		De Korte – de Boer, Dianne
		Buhre, Wolfgang F.F.A.
106	Department of Anesthesiology, Pain Medicine and Procedural Sedation and Analgesia, Martini General Hospital, Groningen, the Netherlands	De Boer, Hans D.
109	Department of Anesthesiology, Medical Center Leeuwarden, Leeuwarden, the Netherlands	Brouwer, Tammo
96	Department of Anesthesiology, NoordWest Ziekenhuisgroep, Alkmaar, the Netherlands	Zwijsen, Johannes H.M.J.
		Edward-Rutten, Gitara M.
98	Department of Anesthesiology, Onze Lieve Vrouwe Gasthuis, Amsterdam, the Netherlands	Godfried, Marc B.
		Thiel, Bram
108	Department of Anesthesiology, Radboud University, Nijmegen, the Netherlands	Hofland, Jan
100	Department of Anesthesiology, Rijnstate, Arnhem, the Netherlands	Ten Hoope, Werner
99	Department of Anesthesiology, Spaarne Gasthuis, Haarlem and Hoofddorp, the Netherlands	Boom, Annemieke
115	Department of Anesthesiology, University Hospital Carl Gustav Carus, Dresden, Germany	Gama de Abreu, Marcelo
119	Department of Anesthesiology, University Hospital of Düsseldorf, Düsseldorf, Germany	Huhn, Ragnar
106	Department of Anesthesiology, University Medical Center Groningen, Groningen, the Netherlands	Struys, Michel M.R.F.
		Zeillemaker-Hoekstra, Miriam
116	Department of Anesthesiology, University of Napoli, Napoli, Italy	Sansone, Pasquale
		Aurilio, Caterina

## Abbreviations

ACS: Acute Coronary Syndrome
AKIN: Acute Kidney Injury Network
ARDS: Acute Respiratory Distress Syndrome
ARISCAT: Assess Respiratory Risk in Surgical Patient in Catalonia
ASA: American Society of Anesthesiologists
BMI: Body Mass Index
COPD: Chronic Obstructive Pulmonary Disease
CRF: Case Report Form
DESIGNATION: Driving prESsure durIng GeNeral AnesThesIa for Open abdomiNal surgery
DSMB: Data Safety Monitoring Board
EPCO: European Perioperative Clinical Outcome
ERAS: Enhanced Recovery After Surgery
FiO_2_: Inspired oxygen fraction
I:E: Inspiratory to Expiratory ratio
ICU: Intensive Care Unit
IRB: Institutional Review Board
MAP: Mean Arterial blood Pressure
NYHA: New York Heart Association
PBW: Predicted Body Weight
PEEP: Positive End-Expiratory Pressure
PPC: Postoperative Pulmonary Complications
RCT: Randomized Clinical Trial
REDCap: Research Electronic Data Capture
RM: Recruitment Maneuvers
TIVA: Total IntraVenous Anesthesia
TOF: Train-Of-Four
VAS: Visual Analog Scale
V_T_: Tidal Volume
ΔP: Driving Pressure
