# Supplementary material for: Driving Pressure During General Anesthesia for Open Abdominal Surgery (DESIGNATION): study protocol of a randomized clinical trial
Source: Trials. 2020 Feb 18;21:198. doi: 10.1186/s13063-020-4075-z (PMC7029544; doi:10.1186/s13063-020-4075-z)
Supplement: Supplementary file 2 — Additional file 2. Model consent form, version 4.0 (11–10-19). [file 13063_2020_4075_MOESM2_ESM.docx]

**Additional file 2** Model consent form, version 4.0 (11-10-19)

**Subject information for participation
in a medical-scientific study**

**Title: Driving pressure during general anesthesia for open abdominal surgery (DESIGNATION) – a randomized clinical trial**

**Introduction**

Dear Sir/Madam,

You have received this letter because you will shortly undergo abdominal surgery. You are being asked to take part in a medical-scientific study. Participation is voluntary. In order to participate your written consent is required. Before you decide whether you want to take part in this study, you will be given an explanation about what the study involves. Please take your time to read this information and ask the investigator if you have any questions. You can also ask the independent expert mentioned at the end of this letter for additional information. You can also discuss it with your partner, friends or family. Further information about participating in such a study is found in the enclosed brochure ‘Medical scientific research’.

1. **General information**

This study was designed by the departments Anaesthesiology and Intensive Care at Amsterdam University Medical Centers (AUMC), location Academic Medical Center (AMC) and is subsidised by the Netherlands Organisation for Health Research and Development (ZonMw). The research is carried out by research physicians in several hospitals in the Netherlands, Italy and Germany. 1468 patients are expected to participate. The Medical Research Ethics Committee of the AUMC, location AMC, has approved this study. You can find general information about the assessment of research in the brochure ‘Medical research’. All contact details of the researchers and the independent physician can be found in **Appendix A**: Contact details.

1. **Purpose of the study**

During an operation under general anaesthesia you will be ventilated by a medical ventilator. Ventilation can have a negative impact on the lungs. Ventilation adjustments have been shown to be beneficial in preventing certain lung complications after surgery. The purpose of this study is to investigate whether a particular ventilation method can prevent these types of complications. To answer that question, we will compare two methods of ventilation. In this study we compare a ventilation method that is commonly used in operations (low airway pressure) with a method with a personalized higher airway pressure. Below we will explain breathing systems in more detail.

**Background of the study**

There are different types of mechanical ventilation. In this study we focus on the following types of ventilation:

- Ventilation with lower pressures. Its disadvantage is that it may lead to the collapse of parts of the lungs. This type of mechanical ventilation is usually used.
- Ventilation with higher pressures. The advantage of this type of ventilation is that the collapse of parts of the lungs is prevented. The disadvantage is that due to higher pressure in the chest, there must be more fluid infusion to improve blood circulation or more medication to prevent lowered blood pressure.

It is currently unclear which type of ventilation is most beneficial to the patient in reducing the risk of lung complications after surgery. In this study, we compare standard ventilation (using low airway pressure) with ventilation with higher airway pressure adapted to the body.

1. **What it means to take part in this study**

**Suitability**

First we will determine whether you are suitable to take part in this study. The researcher will ask you about your medical history and may decide that you cannot take part. Reasons for this may include: you are pregnant, you have COPD for which you receive oxygen therapy at home, you have had an operation on your lungs before, you have a (serious) heart disease, you have been mechanically ventilated in the past 30 days.

**During the operation**

During the operation you will be anaesthetized and you will not notice the type of ventilation used. Just before the operation you will be assigned (by drawing lots) to one of the two ventilation methods. Half of the subjects will be ventilated with high airway pressure adjusted to your body and the other half will be ventilated with the standard low airway pressure. You and the researcher do not know which group you are in. However, if it is important for your health, we can look this up. You can find general information about this in the brochure ‘Medical research’.

**After the operation**

In the days following the operation, a researcher will monitor your health; this is in addition to standard care. After your discharge, you will be called at home two times to find out how you are doing. This will be on day 30 and day 90 after the operation. There is no need to visit the Amsterdam UMC location AMC again for this study.

1. **What is expected of you**

There are no specific requirements for you.

1. **Potential advantages and disadvantages**

It is important that you carefully consider the potential advantages and disadvantages before you decide to participate.

**Advantages**

Participation in the study may be beneficial to you: we assume that ventilation with personalized high airway pressures protects against lung complications in the days following the operation.

**Disadvantages**

However, it cannot be excluded that ventilation with higher pressures may have disadvantages. During the period of ventilation you have a chance of lower blood pressure, which can make it necessary to give you extra medication or fluid to improve blood circulation.

1. **If you do not want to participate, or would like to stop participating in the study**

You decide for yourself whether you want to participate in the study. Participation is voluntary. If you do not want to take part, you will receive the usual ventilation strategy during surgery.

If you do participate in the study, you can always change your mind and decide to stop, at any time during the study except during anesthesia. You do not have to state why you are stopping. However, you should immediately inform the investigator. The data obtained thus far will be used for the study.

1. **End of the study**

Your participation in the study stops when

- you choose to stop
- the investigator considers it best for you to stop
- the Amsterdam UMC, the government or Medical Research Ethics Committee, decides to stop the study.

The study is concluded once all the participants have completed the study. It is expected that the entire study will run for 3 to 4 years. For you as a participant, however, the study is finished after the final telephonic call, which will be conducted 90 days after surgery.

1. **Use and storage of your data**

For this study, your personal data will be collected, used and stored. It involves information such as your name, address, date of birth and data about your health. The collection, use and storage of your data is required in order to answer the questions asked in this study and to be able to publish the results. We ask your consent for the use of your data.

**Confidentiality of your data**

To protect your privacy, your data will receive a code. Your name, date of birth and other information that could directly identify you are therefore omitted. This information can only identify you with the key. The key to the code will be stored securely in the local research facility. The data that is sent to the sponsor only contain a code, but not your name or other data that can identify you. In reports or publications about the study, the data will also not be identifiable.

**Access to your data for review**

Some individuals may have full access to your data at the study site. Also to the data without a code. This is needed in order to check whether the study is performed properly and reliably. Individuals who have access to your data for review are: the study team, the committee monitoring the safety of the study, a monitor that works for the researcher and the Healthcare and Youth Inspectorate. They will keep your data confidential. We ask your consent for this access.

**Retention period of data**

Your data must be stored for 15 years at the study site.

**Storage and use of data for other studies**

Your data may still be of interest after the end of this study for other clinical research in the area of lungprotective ventilation strategies during surgery. For this your data will be stored for 15 years. You can indicate on the consent form if you do or do not agree with this. If you do not consent to this, you can still participate in the current study.

**Withdrawal of consent**

You can always, except during anaesthesia, withdraw your consent for the use of your personal data. This applies to this study. The study data that has been collected until the time you withdraw your consent will still be used in the study.

**More information about your rights concerning the processing of data**

For general information about your rights concerning the processing of your personal data, please consult the website of the Dutch Data Protection Authority.

If you have any questions about your rights, please contact the person responsible for the processing of your personal data. For this study it is:

*Amsterdam University Medical Centers, location Academic Medical Center (AMC)*

*(See contact details in Appendix A).*

If you have any questions or complaints regarding the processing of your personal information, we recommend that you contact the study site. You can also contact the Data Protection Officer for the institution or the Dutch Data Protection Authority. For this study that is:

*Mrs. J.B.Inge,*

*(See contact details in Appendix A)*

**Registration of the study**

Information about this study is also included in a summary of medical research i.e. (clinicaltrial.gov). No data that can be traced back to you is included. After the study, the website may contain a summary of the results of this study. You can find this study under ‘DESIGNATION trial’.

1. **Insurance for subjects**

Insurance has been taken out for everyone who participates in this study. The insurance covers damage resulting from the study. Not all damage is covered. In **Appendix B** you can find more information about the insurance and the exceptions. It also states who you should report damages to.

1. **No compensation for participation**

Participation in the study costs you nothing. You will not be paid for participation in this study.

1. **Do you have any questions?**

If you have any questions, please contact the investigational team. If you would like independent advice about participation in this study, please get in touch with the independent doctor. He knows a lot about the study, but has nothing to do with this study.

If you have any complaints about the study, you can discuss this with the investigator or your regular doctor. If you would rather not do that, you can contact the complaints’ officer at your hospital. All data can be found in **Appendix A**: Contact information.

1. **Signing of informed consent form**

When you have had a sufficient reflection period (at least 12 hours), you will be asked to decide about participation in this study. If you consent, you will be asked to confirm this on the corresponding consent form, in writing. With your written consent, you indicate that you have understood the information and agree to participate in the study.

Both you and the investigator will receive a signed version of this consent form.

Thank you for your attention.

**14. Appendices with this information**

A. Contact details Amsterdam UMC, location AMC

B. Insurance information

C. Informed Consent Form

D. Medical Scientific Research Brochure. General information for Study subjects (version March 2017) **Appendix A: contact details for Amsterdam UMC, location AMC**

| **Principal investigators:** | Prof. dr. Markus W. Hollmann, Anesthesiologist  Department of anesthesiology, Amsterdam UMC location AMC  Meibergdreef 9, H1-132  1105AZ Amsterdam, The Netherlands  Tel. 020 566 3630  Prof. dr. Marcus J. Schultz, Intensivist  Department of Intensive Care, Amsterdam UMC location AMC Meibergdreef 9, C3-423  1105AZ Amsterdam, The Netherlands  Tel. 020 566 2509 |
| --- | --- |
| **Investigators:** | Drs. S.G.L.H. Nijbroek, coordinating investigator  and  Drs. L. Hol, coordinating investigator  Tel. 0205662261 or (81) 58 661 or e-mail: [designation@amc.uva.nl](mailto:designation@amc.uva.nl) |
| **Data protection officer of the institution:** | Mr J.B.M. Inge  Tel. 020 5669111 device number 62015 or e-mail: [j.b.inge@amc.uva.nl](mailto:j.b.inge@amc.uva.nl) |
|  |  |
| **Independent doctor:** | Dr J. Hermanides, Anesthesiologist  Department of anesthesiology, Amsterdam UMC location AMC  Meibergdreef 9, H1-189  1105AZ Amsterdam, The Netherlands  Tel. 020 562 8319 |
| **Complaints:** | Complaints officer AMC Tel. 020 566 3355 |
| **Times of availability**: | Weekdays 9.00 till 15.30 hrs. |

**Appendix B: insurance information**

Insurance has been taken out by Amsterdam UMC for everyone participating in this study. The insurance covers damage due to participation in the study. This applies to damage manifesting during the study or within four years of the end of your participation in the study. You must notify the insurance company about the damage within those four years.

The insurance does not cover all damages. The damages that are not covered are listed briefly at the end of this text.

This is set out in the Medical Research (Human Subjects) Compulsory Insurance Decree. This decree is available (in Dutch) on [www.ccmo.nl](http://www.ccmo.nl), the website of Central Committee on Research Involving Human Subjects (see ‘Bibliotheek’ en dan ‘Wet- en regelgeving’).

In the event of damage please contact the insurance company directly.

The insurance company for the study is:

Name: Centramed B.A.

Address: Postbus 7374

2701 AJ Zoetermeer

Telephone number: 070 301 70 70

E-mail: [info@centramed.nl](mailto:info@centramed.nl)

Policy number: 624.528.303

The insurance offers a cover of € 650,000 per study subject and a maximum of € 5,000,000 for the entire study and € 7,500,000 annually for all studies from the same sponsor.

The insurance policy does **not** cover the following damage:

- damage as a result of a risk that you were informed about in the written information. This does not apply if the risk occurs in a more severe form than envisaged, or if the risk was very unlikely to occur;
- damage to your health that would also have occurred if you had not participated in the study;
- damage resulting from not or not entirely following directions or instructions;
- damage to descendants as a result of a negative effect of the study on you or your descendants;
- damage as a result of an existing treatment method for research into existing methods of treatment

In case of damage, the participant of the study should first contact Prof.dr. Markus W. Hollmann ( 020 566 3630) or Prof. dr. Marcus J. Schultz (020 566 2509)

**Appendix C: Subject Consent Form**

**Airway pressure during general anesthesia for abdominal surgery**

- I have read the information letter. I was also able to ask questions. My questions have been answered sufficiently. I have had enough time to decide whether or not to participate.
- I understand that participation is voluntary. I also know that I may decide at any time to not participate or to stop participating in the study. Without having to provide any reason.
- I give consent for my treating specialist(s) to be informed of my participation in this study.
- I give consent to collect and use my data for answering the research question in this study.
- I know that for study monitoring purposes some individuals could have access to all my data. Those people are listed in this information letter. I consent to that access by these persons.
- I □ **give**

□ **do not give**
consent for the further storage of my personal data and retention for future research into the area of protective mechanical ventilation strategies during surgery.

- I □ **give**

□ **do not give**consent to being contacted again after this study for a follow-up study.

- I want to participate in this study.

Name of subject: ……………………………………………………………………………..

Signature: Date : __ / __ / __

-----------------------------------------------------------------------------------------------------------------

I certify that I have fully informed this subject about the said study.

If information becomes known during the study that could influence the consent of the subject, I will inform him/her of this on time.

Name of investigator (or his/her representative):…………………………………………

Signature: Date: __ / __ / __

-----------------------------------------------------------------------------------------------------------------

*The subject will receive a complete information letter, together with a signed version of the informed consent form.*
